# Navigating Outcomes and Tailored Treatment Choices in a Cesarean Scar Ectopic Pregnancy: Insights From Five Cases in a Resource-Limited Facility

**DOI:** 10.7759/cureus.118308

**Published:** 2026-10-21

**Authors:** Dancy Patel, Saunri Hansadah, Sonali Jagati

**Affiliations:** 1 Obstetrics and Gynaecology, Srirama Chandra Bhanja (SCB) Medical College and Hospital, Cuttack, IND

**Keywords:** av malformation, caesarian scar, cesarean scar ectopic pregnancy, cesarean section, internal iliac artery ligation, methotrexate, scar excision, scar site pregnancy, suction evacuation, ultrasonography (usg)

## Abstract

Once considered a rare entity, cesarean scar pregnancy (CSP) is now increasingly encountered. However, relatively little is known about its clinical characteristics and management due to the lack of large population-based studies. It results from implantation of a gestational sac within the fibrous scar of a previous cesarean section. Despite increasing awareness, there is no standardized management protocol, which can lead to delayed diagnosis and considerable variation in treatment approaches and outcomes. We report five cases of CSP managed at a tertiary care center between 2023 and 2024. The patients were 27 to 38 years of age, had one to two previous cesarean deliveries, and presented at gestational ages ranging from seven to nearly 17 weeks. Initial presenting complaints included vaginal bleeding, passage of a fleshy mass, and abdominal pain. Two patients had used over-the-counter medical termination kits, and one had a history of failed suction and evacuation. The diagnosis was established primarily by ultrasonography, with serum beta-human chorionic gonadotropin (hCG) used to support the diagnosis and monitor treatment response. Management was individualized: One patient was initially treated with methotrexate and mifepristone but required emergency laparotomy because of persistent vaginal bleeding; another underwent scar site resection and repair after hemodynamic stabilization; a third, with suspected arteriovenous malformation, required bilateral uterine and internal iliac artery ligation before scar excision; a fourth underwent hysterectomy; and the fifth underwent scar excision with concurrent bilateral tubal ligation. There were no maternal deaths, and all patients were discharged in stable condition. These cases demonstrate the varied clinical presentations and management challenges associated with CSP, including misdiagnosis and the need for individualized treatment ranging from conservative, fertility-preserving surgery to hysterectomy. CSP can mimic incomplete abortion and, if misdiagnosed and treated with unsupervised medical termination kits, can lead to catastrophic hemorrhage and potentially fatal outcomes. Therefore, obstetricians should maintain a high degree of suspicion for scar ectopic pregnancy in women with a history of cesarean section who present with early pregnancy bleeding. Early diagnosis and individualized, timely management - medical, surgical, or a combination of both - can achieve satisfactory maternal outcomes and, in many cases, preserve future fertility.

## Introduction

Hidden within the scar of a previous cesarean section lies one of the rarest forms of ectopic pregnancy, cesarean scar pregnancy (CSP), which has emerged as an increasingly recognized complication in modern obstetrics, affecting approximately five to six per 10,000 pregnancies [1]. This estimate may be an underestimate due to the lack of large population-based studies, and reported incidence rates have therefore varied across different literature reviews.

CSP can be defined as a form of ectopic pregnancy in which the gestational sac implants within the fibrous scar of a previous cesarean section [2]. Recent classification systems have described CSP beyond the traditional endophytic (type 1) and exophytic (type 2) types [3], incorporating ultrasound features such as residual myometrial thickness, degree of gestational sac protrusion, presence of fetal cardiac activity, and vascularity on Doppler ultrasound to predict clinical outcomes and guide management [4]. Various treatment modalities are available; however, the absence of a standardized protocol often requires obstetricians to tailor medical, surgical, or expectant management to individual cases. Given the uncertainty surrounding optimal management, further studies are needed.

To add value to our understanding of CSP, we present five cases of CSP in patients who presented to our center during 2023-2024 with various clinical complaints, diagnostic journeys, and were managed using different clinical approaches.

## Case presentation

Case 1

A 38-year-old pregnant woman, G2P1L1, with one previous cesarean delivery, presented with amenorrhea for eight weeks and two episodes of vaginal bleeding. She had severe anemia and presented for medical termination of pregnancy (MTP). On examination, she was significantly pale but had stable vital signs. The abdomen was soft and nontender. On per vaginal examination, the uterus was anteverted, firm, mobile, and approximately six to eight weeks in size. The cervix was soft, and the os was closed. Ultrasonography showed a gestational sac with a live fetus in the lower uterine segment, with a crown-rump length corresponding to seven weeks and four days. The gestational sac was located within the endometrium, with surrounding myometrium < 5 mm at the scar site (Figure 1A). The beta-human chorionic gonadotropin (hCG) level was 108,600 IU/L, and hemoglobin was 6.3 g/dL. Routine investigations were performed, and three units of blood were transfused. A provisional diagnosis of scar site pregnancy was made, and she was administered methotrexate 50 mg along with oral mifepristone 200 mg. After three days, the beta-hCG level had decreased only minimally to 98,000 IU/L, and she experienced an episode of severe bleeding, for which laparotomy was performed. The site of the scar ectopic pregnancy was identified intraoperatively (Figure 1B). An incision was made above the previous scar site, and the products of conception were evacuated (Figure 1C). The anterior scar site was subsequently repaired (Figure 1D). Intraoperatively, one unit of packed red blood cells was transfused. The postoperative period was uneventful, and she was discharged on postoperative day 7 following skin suture removal.

**Figure 1:**
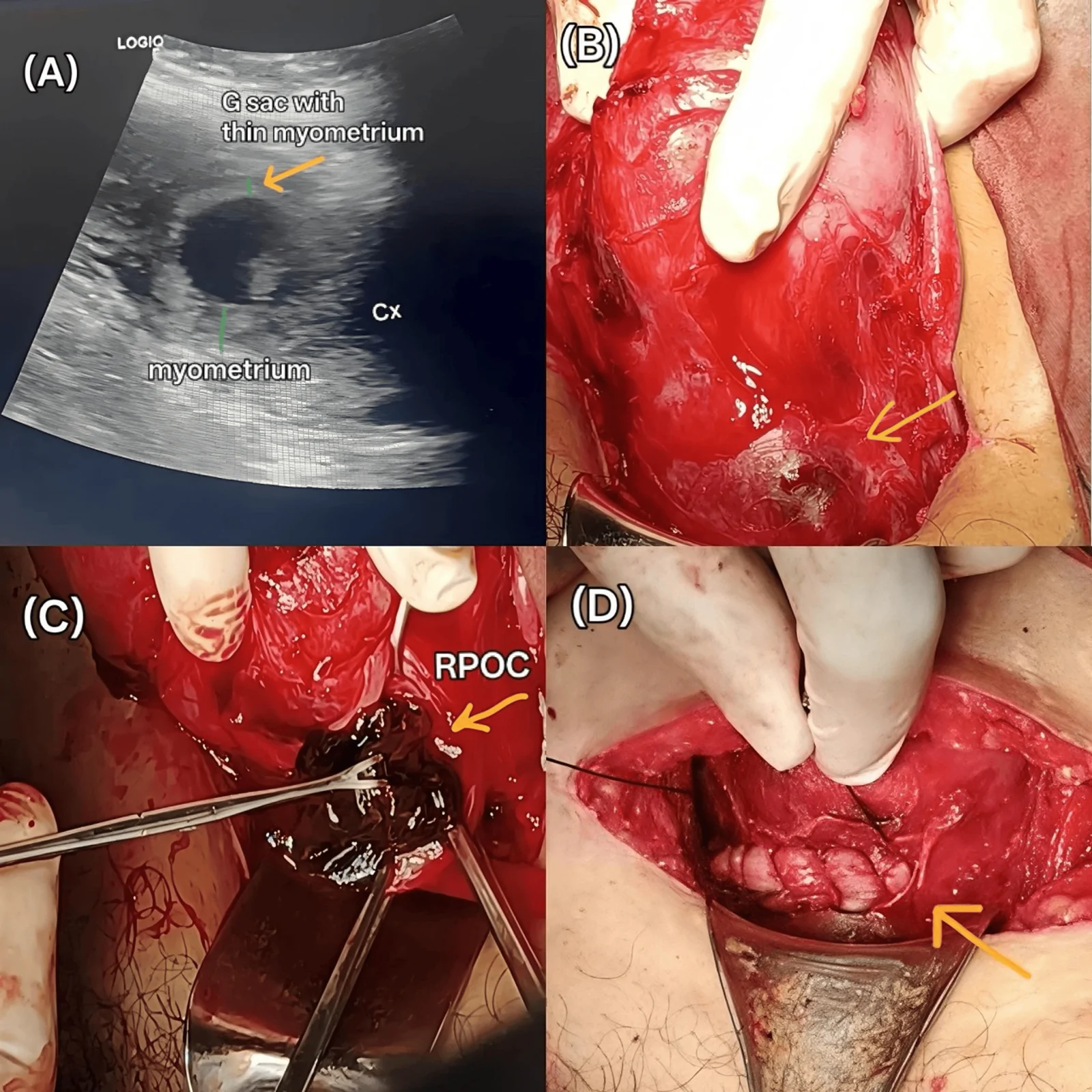
Cesarean scar site ectopic pregnancy of case 1. (A) Ultrasonography showing gestational sac and fetal pole (arrow). (B) Intraoperative picture of scar site pregnancy (arrow). (C) Products of conception after scar site is incised (arrow). (D) Scar excision and repair of anterior wall of uterus (arrow).

Case 2

A 32-year-old woman, G3P2L2, with two previous cesarean deliveries and 12 weeks of amenorrhea, was admitted with complaints of vaginal bleeding and passage of a fleshy mass for one day after taking an oral abortifacient. On examination, she was in shock. The abdomen was soft, with diffuse tenderness. The uterus was approximately 14 weeks in size, and both fornices were tender. The cervical os was open, and approximately 50 mL of clots were spontaneously expelled through the os. The patient was transfused with two units of packed red blood cells and started on vasopressors. Her serum beta-hCG level was 17,660 mIU/mL. Because of the unclear diagnosis and misleading clinical findings, she was first stabilized in the high-dependency unit. A repeat beta-hCG level was 5,620 mIU/mL, and a second opinion on ultrasonography showed a heterogeneous echogenic lesion with internal vascularity in the lower endometrial cavity in the cervical region, raising suspicion of a scar ectopic pregnancy (Figure 2A). The sliding sign was negative. She subsequently underwent laparotomy, during which an endophytic scar site ectopic pregnancy was identified (Figure 2B) and resected, followed by repair of the uterus. She was discharged with stable vital signs on postoperative day 5.

**Figure 2:**
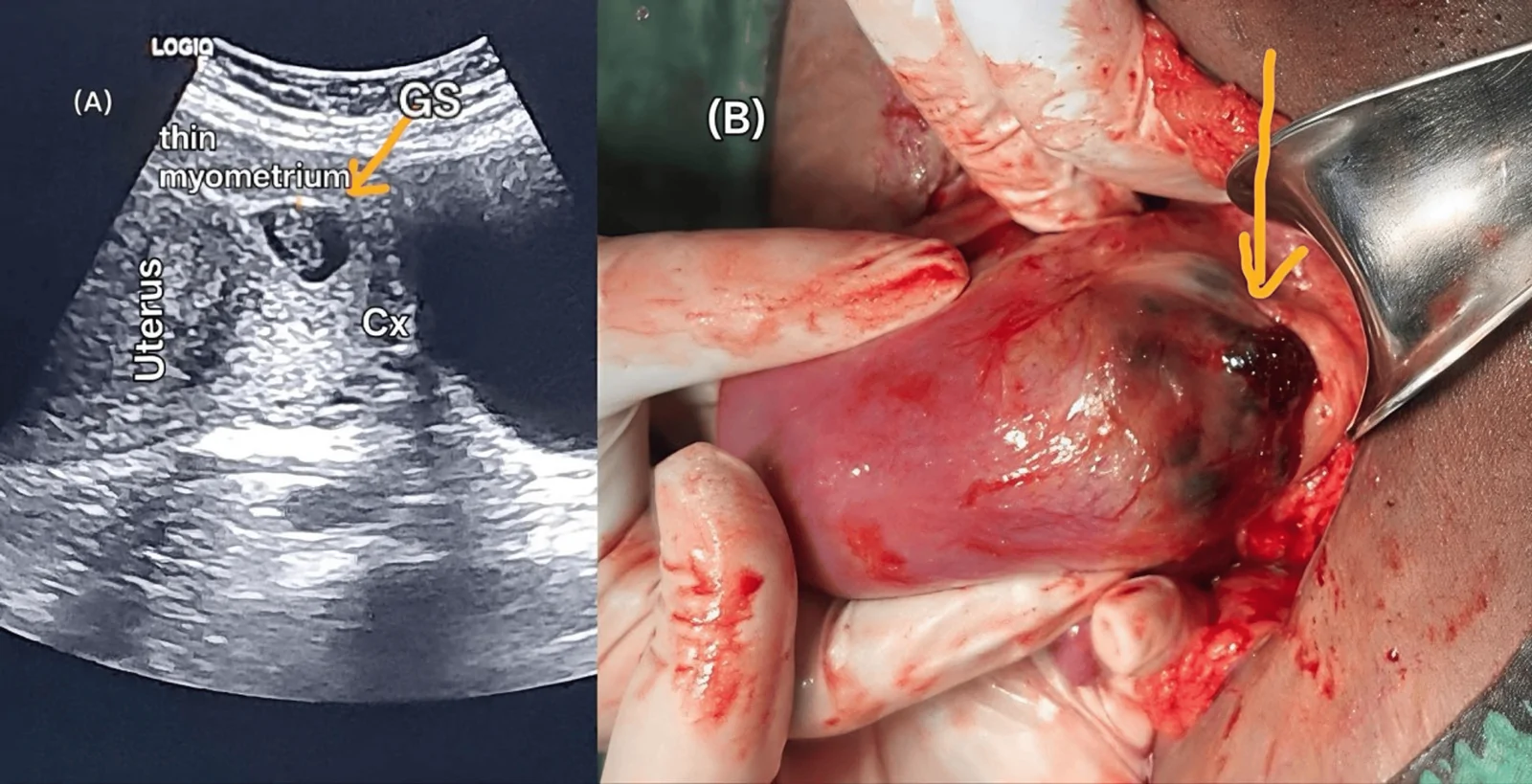
Cesarean scar site pregnancy of case 2. (A) Grey scale ultrasonography showing implantation at lower uterine segment near cervical region (arrow). (B) Intraoperative image of the scar site pregnancy (arrow).

Case 3

A 27-year-old woman, G4P1L1A2, with one previous cesarean delivery, was admitted with a chief complaint of intermittent vaginal bleeding for 20 days after 14 weeks of amenorrhea. For this, she had undergone suction and evacuation twice, with the most recent procedure performed 20 days earlier. On admission, her shock index was 1.2. The abdomen was soft and non-tender. On per speculum examination, there was no active bleeding. On per vaginal examination, the uterus was approximately eight to 10 weeks in size and mobile, and both fornices were free. Ultrasonography showed multiple irregular, echogenic tubular spaces in both the anterior and posterior myometrium with significant vascularity on color Doppler, raising suspicion of an arteriovenous malformation (AVM) (Figures 3A-3B). Her serum beta-hCG level was 132 mIU/mL. She underwent laparotomy. On opening the abdomen, the uterus was found to be approximately 12 weeks in size, with hypervascularity and tortuous vessels over the anterior lower uterine segment and bladder surface (Figure 3D). At the previous scar site in the lower uterine segment, ectopic gestational tissue was identified (Figure 3C). Bilateral uterine artery ligation, ligation of the utero-ovarian anastomoses, and internal iliac artery ligation were performed, followed by excision of the scar site pregnancy. She received two units of packed red blood cells and was discharged with stable vital signs. Histopathological examination of the excised tissue showed retained products of conception.

**Figure 3:**
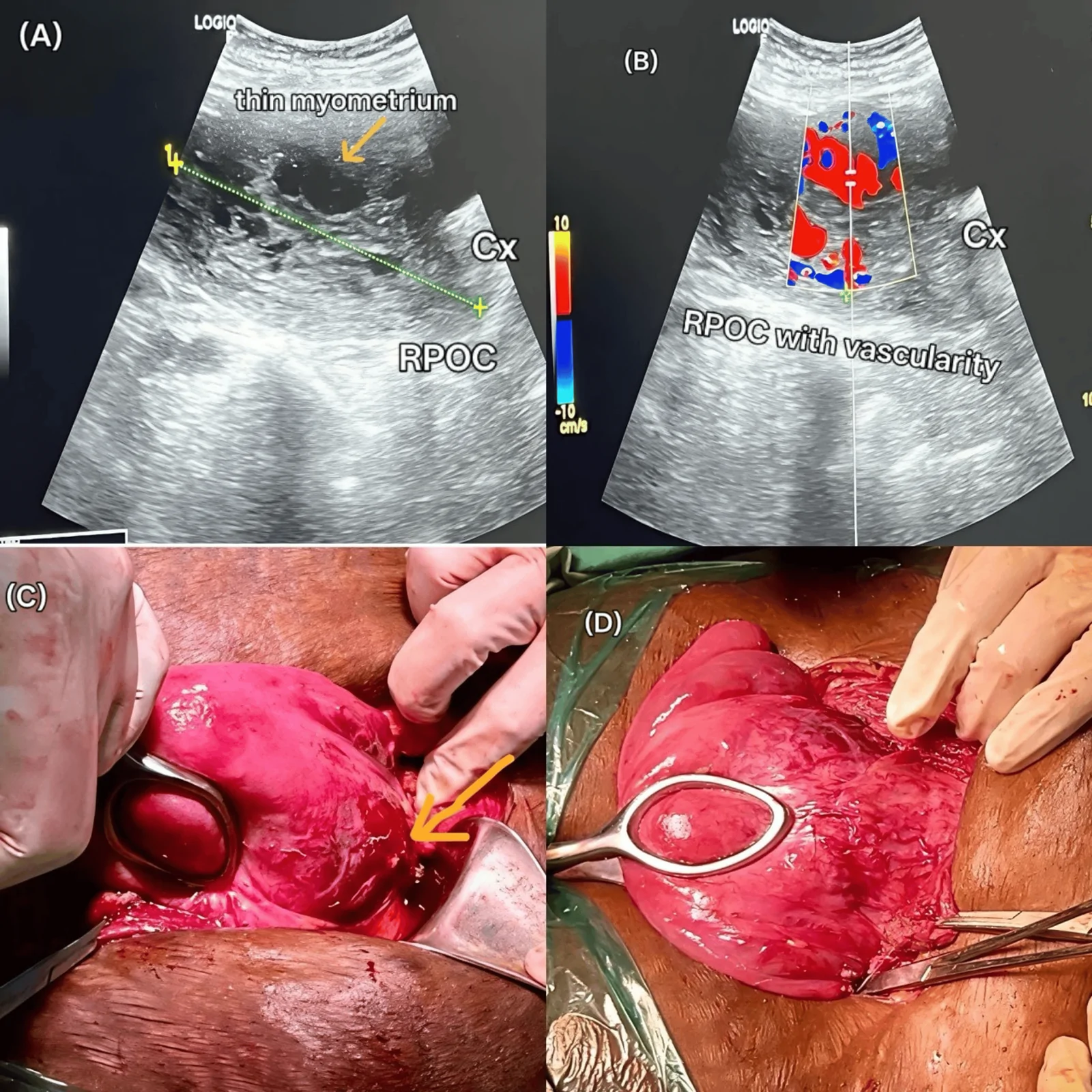
Cesarean scar site pregnancy of case 3, associated with arteriovenous malformation (AVM). (A) Gray scale ultrasonography showing multiple irregular and echogenic tubular spaces in both anterior and posterior myometrium. (B) Vascularity seen on color Doppler. (C) Intraoperative image of scar site pregnancy (arrow). (D) Intraoperative image showing tortuous vessels of associated AVM.

Case 4

A 35-year-old woman, G3P2L2, with two previous cesarean deliveries and seven weeks of amenorrhea, was admitted with complaints of abdominal pain and vaginal spotting for two days. On examination, her vital signs were stable, and the abdomen was soft with mild tenderness. On vaginal examination, the uterus was bulky, with no forniceal tenderness. Ultrasonography showed a gestational sac at the scar site with a fetal pole but no cardiac activity. She was planned for laparotomy with consent for hysterectomy. On opening the abdomen, a thin uterine scar with a scar-site ectopic pregnancy was identified (Figure 4A). She underwent hysterectomy without complications (Figures 4B-4C). She was discharged with stable vital signs after five days.

**Figure 4:**
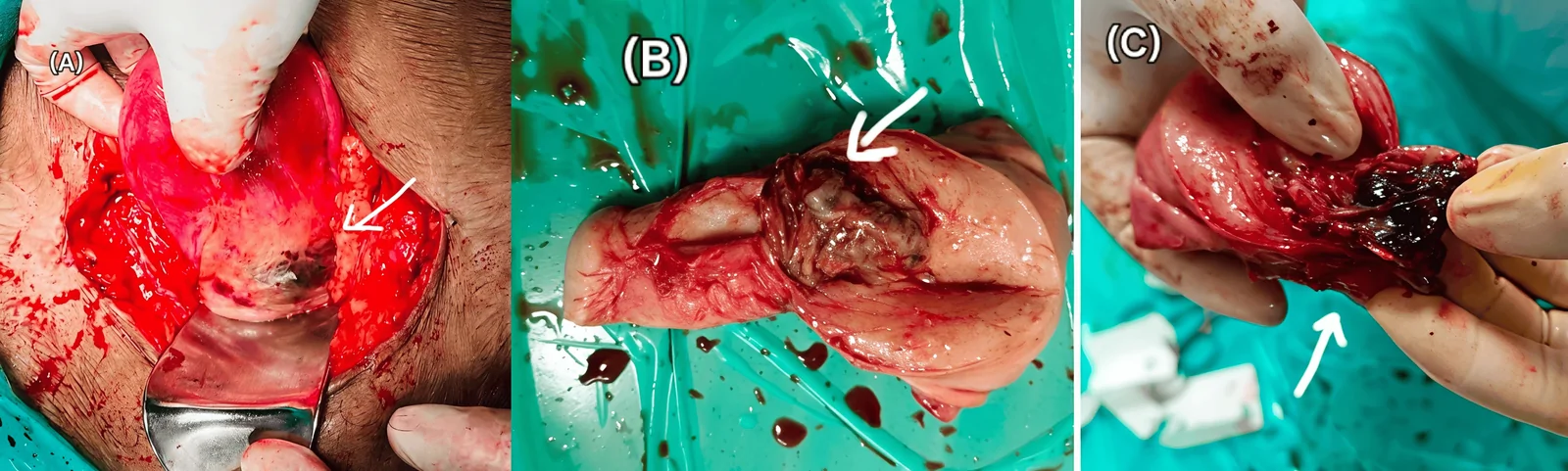
Cesarean scar site pregnancy of case 4. (A) Intraoperative image of scar site pregnancy (arrow). (B) Total hysterectomy sample with scar ectopic pregnancy (arrow). (C) Cut section of uterus showing endophytic scar ectopic pregnancy.

Case 5

A 35-year-old woman, G3P2L1, with two previous cesarean deliveries, was admitted with complaints of vaginal bleeding for two months and passage of a fleshy mass two months earlier. Two months earlier, following 16 weeks and six days of amenorrhea, she had taken an oral abortifacient after a self-administered urine pregnancy test was positive. On admission, she was hemodynamically stable. The abdomen was soft and non-tender, and on pelvic examination, the uterus was normal in size. Her serum beta-hCG level on admission was 23.18 mIU/mL. Ultrasonography showed a hyperechoic area measuring 2.2 × 1.78 cm in the anterior myometrium of the lower uterine segment at the scar site, with marked vascularity, suggestive of retained products of conception and raising suspicion of a scar site ectopic pregnancy (Figure5A). Laparotomy was performed, and an ectopic mass measuring 4 × 3.5 cm was identified at the cervico-isthmic junction. A transverse incision was made over the mass, which was removed, and the anterior uterine wall was repaired (Figures 5B-5C). The tissue sample was sent for histopathological examination. Bilateral tubal ligation was also performed. On follow-up, the serum beta-hCG level decreased to 2.11 mIU/mL. Histopathological examination showed products of conception, confirming the diagnosis of scar site ectopic pregnancy, while examination of the tubal specimens showed salpingitis. She was discharged with stable vital signs after 10 days.

**Figure 5:**
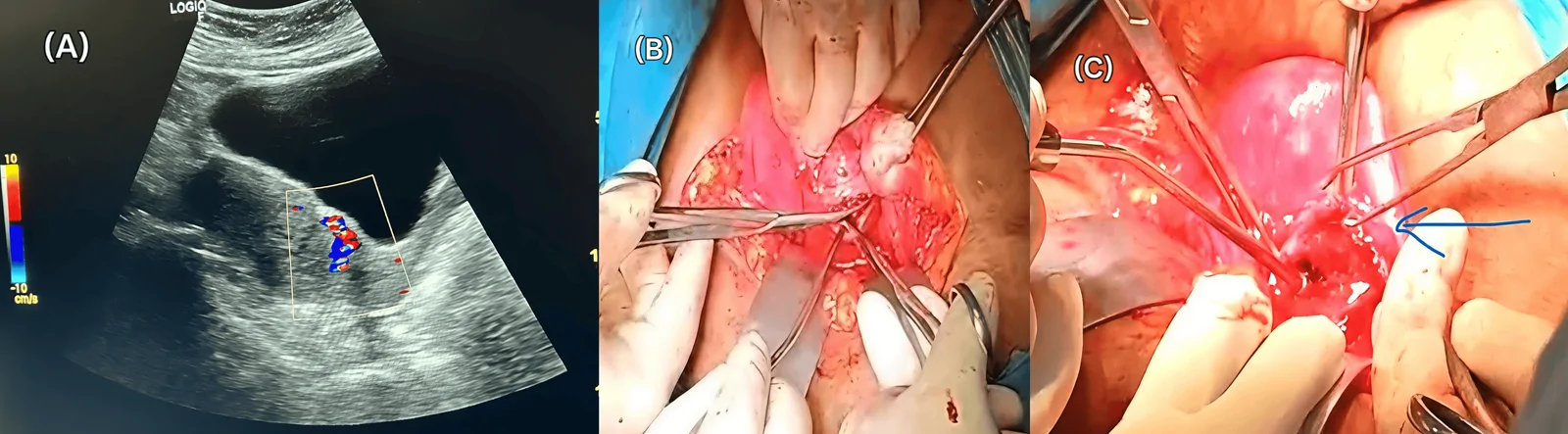
Cesarean scar site pregnancy of case 5. (A) Ultrasonography showing hyperechoic area in anterior myometrium at scar site with high vascularity suggestive of scar site ectopic pregnancy (box). (B) Intraoperative image showing incision being made at scar site. (C) Edges of scar site held for repair after removing products of conception (arrow).

All five cases are summarized in Table 1, comparing patient age, parity, gestational age at presentation, diagnostic findings, adverse and favorable outcomes, and treatment modalities.

**Table 1:** Summary of case reports of all five cases. BTL, bilateral tubal ligation; CPD, cephalopelvic disproportion; CS, cesarean section; GS, gestational sac; MTP, medical termination of pregnancy; NPL, non-progress of labor; RPOC, retained products of conception

	Case 1	Case 2	Case 3	Case 4	Case 5
Age	38 years	32 years	27 years	35 years	35 years
Previous CS	1 (G2P1L1)	2 (G3P2L2)	1 (G4P1L1A2)	2 (G3P2L2)	2 (G3P2L1)
Gestational age	8 weeks	12 weeks	14 weeks	7 weeks	16 weeks 6 days
Presenting complaints	Vaginal bleeding and abdominal discomfort	Vaginal bleeding and passage of a fleshy mass	Vaginal bleeding	Abdominal pain and vaginal spotting	Vaginal bleeding for 2 months
Indication for previous CS	Oligohydramnios	NPL	CPD	Unknown	CPD
Previous pregnancy complications	Uneventful	Uneventful	Uneventful	Unknown	Uneventful
Used MTP kit	No	Yes	No	No	Yes
Ultrasonography findings	GS and fetal pole with cardiac activity located within the endometrium with surrounding myometrium at the scar site	Heterogeneous mixed echogenic lesion with internal vascularity in the lower endometrial cavity	RPOC with suspected arteriovenous malformation	Fetal pole without cardiac activity	Products of conception in the anterior myometrium of the lower uterine segment at the scar site
Type of CSP	Endophytic growth	Endophytic growth	Exophytic growth	Endophytic growth	Exophytic growth
Primary treatment	Methotrexate injection followed by mifepristone	Scar site resection and repair after stabilization	Suction and evacuation	Hysterectomy	Scar excision and repair with BTL
Secondary treatment	Laparotomy with scar excision and repair	None	Laparotomy with devascularization, scar resection, and repair	None	None

## Discussion

Approximately 3-6% of pregnancy-related deaths are attributed to ectopic pregnancies [5]. Although the reported rate of cesarean deliveries has increased, CSP remains one of the rarest forms of ectopic pregnancy. As demonstrated by our cases, it can occur regardless of the number of previous cesarean deliveries. Diagnosis of scar ectopic pregnancy can be challenging because it may closely mimic a miscarriage, particularly in patients presenting with vaginal bleeding.

Of the five cases, two patients had taken over-the-counter MTP kits, and one had a history of failed suction and evacuation. With the widespread use of ultrasonography, approximately 48% of cases are diagnosed incidentally during first-trimester scans, while approximately 33% present with painless vaginal bleeding and 19% with abdominal pain [6]. In our case series, three patients presented with painless vaginal bleeding, while two had associated abdominal pain. This difference may be attributable to the fact that our cases were managed at a tertiary care center and that some patients presented after consuming MTP kits. Although the pathogenesis is not completely understood, abnormal implantation of a blastocyst through a defect in a previous cesarean section scar is thought to result from defective decidualization [7] and the formation of a niche that facilitates abnormal trophoblastic invasion into the myometrium [8]. Several risk factors have been proposed to increase the incidence of CSP, including cesarean delivery during the second stage of labor, a retroflexed uterus, gestational diabetes, increased maternal body mass index, perioperative fever or infection, and previous myomectomy [9]. These factors may predispose women to persistent uterine scar defects following cesarean delivery. Accordingly, an “endometrial-free” uterine incision closure technique has been associated with reduced niche formation in several studies [10]. However, a recent systematic review reported that the incidence of scar site ectopic pregnancy was similar with single- and double-layer uterine closure and was not significantly affected by the number of previous cesarean sections [11].

The differential diagnoses of CSP include cervical pregnancy, failed intrauterine pregnancy, and miscarriage in progress, which could be differentiated based on residual myometrial thickness, color Doppler flow, and the sliding sac sign. Although there are no universally accepted diagnostic criteria for scar site ectopic pregnancy, ultrasonography is the primary diagnostic modality, with characteristic features including an empty uterine and cervical canal and a thin residual myometrial layer, usually less than 5 mm [12]. Endophytic and exophytic growth of scar site pregnancy can be classified ultrasonographically by residual myometrial thickness and corroborated with intravenous-operative findings [13]. Magnetic resonance imaging may be used as an adjunct to further support the diagnosis. Serum beta-hCG levels are not diagnostic but form part of some proposed post-treatment follow-up protocols, which include weekly beta-hCG measurements until the level becomes undetectable, along with ultrasonographic monitoring [14].

Multiple treatment modalities have emerged in recent years; however, there is still no standardized protocol for optimal management. Expectant management requires careful patient counseling regarding the risk of rupture and potentially life-threatening hemorrhage. Medical management includes methotrexate administered systemically or intragestational sac injection and other intra-sac injection of embryotoxic agents such as potassium chloride, with appropriate preparation for emergency laparotomy if required. Although single-dose therapy may be effective in some cases, multiple doses or subsequent surgical intervention may be necessary. One study reported a 74% success rate with combined local and systemic methotrexate therapy [15]. Methotrexate may be combined with other proposed adjunctive medications like oral mifepristone [16], which was followed in our first case.

Surgical management includes dilation and curettage with preparedness for significant hemorrhage, scar excision and repair with preservation of the uterus and fertility, or hysterectomy based on the clinical circumstances and patient’s preferences [17]. Scar excision is generally considered when the pregnancy is exophytic, whereas suction evacuation may be considered for endophytic scar ectopic pregnancy when adequate residual myometrial thickness is present [18]. In our resource-limited facility, interventional radiological modalities, fetal medicine specialists, and advanced minimal access surgical procedures are unavailable. Hence, in our case series, one patient was initially treated with systemic methotrexate and mifepristone as she was vitally stable and preferred conservative management but subsequently required laparotomy. Most patients were successfully managed with scar excision and repair. In one case, the scar site pregnancy was associated with abnormal vascularity and suspected AVM, necessitating pelvic devascularization before excision. Though the five cases had immediate successful outcomes, long-term follow-up could not be done to assess the overall fertility outcomes. Timely diagnosis and an individualized treatment plan may reduce the risk of severe maternal morbidity and mortality associated with CSP [19].

## Conclusions

Although CSP is a rare form of ectopic pregnancy, it is being increasingly recognized in clinical practice. However, several studies are required to standardize the diagnostic and treatment protocols. With the widespread use of ultrasonography, it may be detected incidentally and can be successfully managed with individualized medical or surgical treatment, including scar excision and repair or hysterectomy when indicated. It is important to consider scar site pregnancy in patients with a previous cesarean delivery who present with early pregnancy bleeding, as it can mimic incomplete abortion but may result in life-threatening hemorrhage and maternal mortality if misdiagnosed or inadequately managed. Although CSP can pose significant diagnostic and management challenges, timely diagnosis and appropriate individualized treatment can result in satisfactory maternal outcomes.
